# Use of a graph neural network to the weighted gene co-expression network analysis of Korean native cattle

**DOI:** 10.1038/s41598-022-13796-9

**Published:** 2022-06-14

**Authors:** Hyo-Jun Lee, Yoonji Chung, Ki Yong Chung, Young-Kuk Kim, Jun Heon Lee, Yeong Jun Koh, Seung Hwan Lee

**Affiliations:** 1 Department of Bio-AI Convergence, Chungnam National University, Daejeon, 305-764 Korea; 2 Division of Animal and Dairy Science, Chungnam National University, Daejeon, 305-764 Korea; 3 Department of Beef Science, Korea National College of Agriculture and Fisheries, Wanju, 54874 Korea; 4 Department of Computer Science & Engineering, Chungnam National University, Daejeon, 305-764 Korea

**Keywords:** Bioinformatics, Biological models, Gene expression analysis

## Abstract

In the general framework of the weighted gene co-expression network analysis (WGCNA), a hierarchical clustering algorithm is commonly used to module definition. However, hierarchical clustering depends strongly on the topological overlap measure. In other words, this algorithm may assign two genes with low topological overlap to different modules even though their expression patterns are similar. Here, a novel gene module clustering algorithm for WGCNA is proposed. We develop a gene module clustering network (gmcNet), which simultaneously addresses single-level expression and topological overlap measure. The proposed gmcNet includes a “co-expression pattern recognizer” (CEPR) and “module classifier”. The CEPR incorporates expression features of single genes into the topological features of co-expressed ones. Given this CEPR-embedded feature, the module classifier computes module assignment probabilities. We validated gmcNet performance using 4,976 genes from 20 native Korean cattle. We observed that the CEPR generates more robust features than single-level expression or topological overlap measure. Given the CEPR-embedded feature, gmcNet achieved the best performance in terms of modularity (0.261) and the differentially expressed signal (27.739) compared with other clustering methods tested. Furthermore, gmcNet detected some interesting biological functionalities for carcass weight, backfat thickness, intramuscular fat, and beef tenderness of Korean native cattle. Therefore, gmcNet is a useful framework for WGCNA module clustering.

## Introduction

Weighted gene co-expression network analysis (WGCNA) is often used to explore the system-level functionality of gene sets. WGCNA groups thousands of genes into a number of modules, simplifying biological interpretation. The general framework of WGCNA^1^ can be summarized as follows. First, the adjacencies of paired genes are calculated to define the gene co-expression network. The adjacencies are then incorporated into a topological overlap measure (TOM) to reveal gene-gene connections. Using the TOM, a clustering algorithm assigns intensively connected genes to the same modules. Finally, functional analyses are used to determine the biological meanings of the modules. This pipeline has been widely used in various fields. For example, recent biomedical studies used WGCNA to identify specific modules and hub genes related to human cancer^2^ and arterial disease^3^. In animal and plant sciences, WGCNA has often been used to profile plant gene expression^4^ and detect pathways responsible for complex animal traits^5,6^. The module definitions greatly affect the interpretations of the results. WGCNA commonly uses a hierarchical clustering (HC) algorithm. This unsupervised clustering method places adjacent genes into the same modules based on pairwise TOM data. However, a concern has been raised that transformations of gene expressions into a TOM results in loss of raw-level expression features. HC-based module assignment depends strongly on the TOM. This can degrade similarity of expression not only between modules, but also within modules. In other words, HC may assign two genes with low topological overlap to different modules even though their expression patterns are similar. Furthermore, once a gene is added to a specific module, HC can never reverse the decision. This poses challenges when clustering complicated networks with many interconnected gene pairs. Thus, a new algorithm is needed to more accurately identify WGCNA gene modules. Langfelder et al.^7^ developed a “dynamic tree cut” technique that clusters gene modules based on the shapes of dendrogram branches, but this still depends on TOM. Botía et al.^8^ employed a derivative of K-means processing to refine gene modules generated by standard HC. However, this algorithm requires more than four steps beginning with module clustering, centroid computation, distance measurements, and gene relocation. This complex pipeline requires significant computational time and is thus unsuitable for very large networks.

A graph neural network (GNN)^9–12^ is a good alternative algorithm for module clustering. GNNs extend deep neural networks to learn a graph representation by finding stable features of nodes and its neighbors in graph-based data. Gilmer et al.^11^ introduced a general framework termed message-passing neural network (MPNN), which effectively aggregates each node with its neighbors into embedding features. Many other studies for GNN have achieved impressive performance using this framework^12,13^. Given the recent successes of GNN, graph-based learning methods have been widely applied in bioinformatics. To predict drug-target interactions, recent studies employed various graphical convolutional networks^14,15^. For single-cell RNA-seq analysis, a GNN was used to model cell-cell relationships^16^ and impute gene expression levels within single cells^17^. Yang et al.^18^ developed a GNN that extracted protein features from graphical information. However, most studies on WGCNA did not use GNN for module clustering.

In this paper, we introduce a GNN-based clustering algorithm for WGCNA: the gene module clustering network (gmcNet). Our method clusters genes based on their co-expression topologies (genes in the same module should be strongly connected) and single-level expression (genes in the same module should exhibit similar expression patterns). The main innovation of gmcNet is incorporating the expression feature of single gene with co-expression feature of their neighbor genes. gmcNet includes a “co-expression pattern recognizer” (CEPR) and a module classifier. The CEPR has a message-passing (MP) operation similar to that of MPNN^11^, except that the topological overlap matrix^1^ is used as the input rather than the adjacency matrix. Using the former matrix, CEPR defines weighted relationships, consistent with the objective of WGCNA. The module classifier assigns genes to various modules using the CEPR-embedded features. We tested gmcNet using RNA-seq data for native Korean cattle, and compared the performance to that of other clustering algorithms. We also validated gmcNet performance on gene expression datasets of human, mouse, pig, and chicken which were downloaded from the Gene Expression Omnibus (GEO) repository^19^. As GNNs are not widely used for WGCNA, our findings will be of interest to computational biologists.

**Figure 1:**
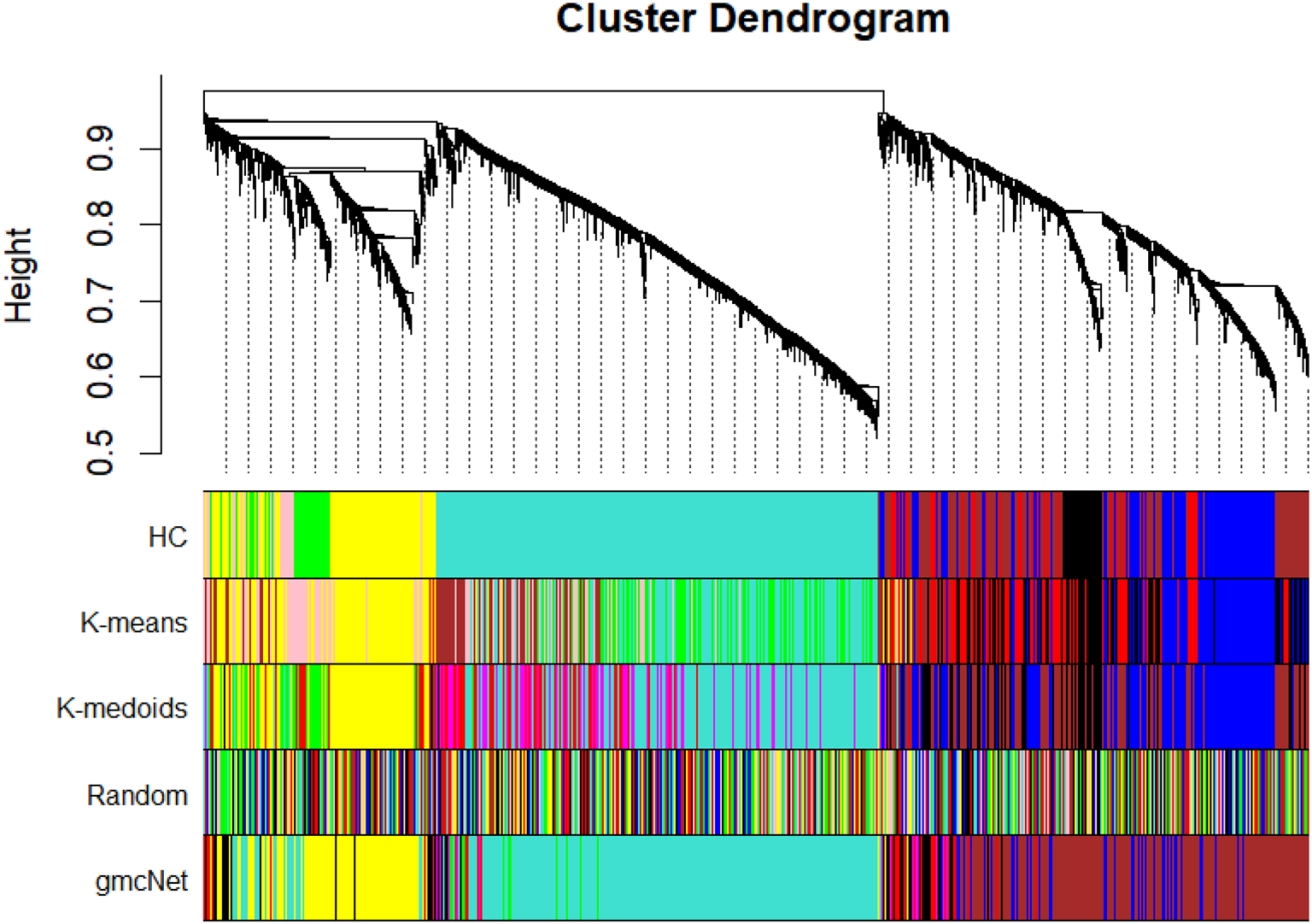
Module clustering results. The upper panel displays the hierarchical clustering dendrogram. In the lower panel, the colors show the module memberships determined by the methods on the left.

## Results

### Model performance on Korean native cattle

To validate gmcNet performance, it was compared to four baseline clustering algorithms including HC, K-means clustering, and K-medoids clustering (Fig. 1). We measured performance in terms of clustering strength and functional enrichment. We used graph modularity^20^ to measure the clustering strength, and the differentially expressed module (DEM) signals to assess functional enrichment.

Table 1 presents the performances of the various methods on Korean native cattle dataset. The single gene expression-based method (K-means) is robust to DEM signal capture, whereas the TOM-based methods (HC, K-medoids) provide higher modularity. On the other hand, gmcNet, which leverages both single gene expression and TOM, achieves the best DEM signal (27.739) and cluster modularity ($\mathcal {Q}$: 0.261). Comparison of gmcNet and HC revealed that gmcNet markedly increases modularity and the DEM signal by 0.042 and 9.121, respectively. Thus, gmcNet is more powerful than the other methods for revealing the apparent closeness of genes within the same module, and when making biological sense of the complex traits of native Korean cattle.

**Table 1:** Model performance on Korean native cattle dataset in terms of graph modularity $\mathscr{Q}$ and DEM signaling.

Method	HC	K-means	K-medoids	gmcNet
$\mathscr{Q}$	0.219	0.138	0.171	0.261
DEM-signal	18.618	22.723	18.236	27.739

### CEPR embedding

**Figure 2:**
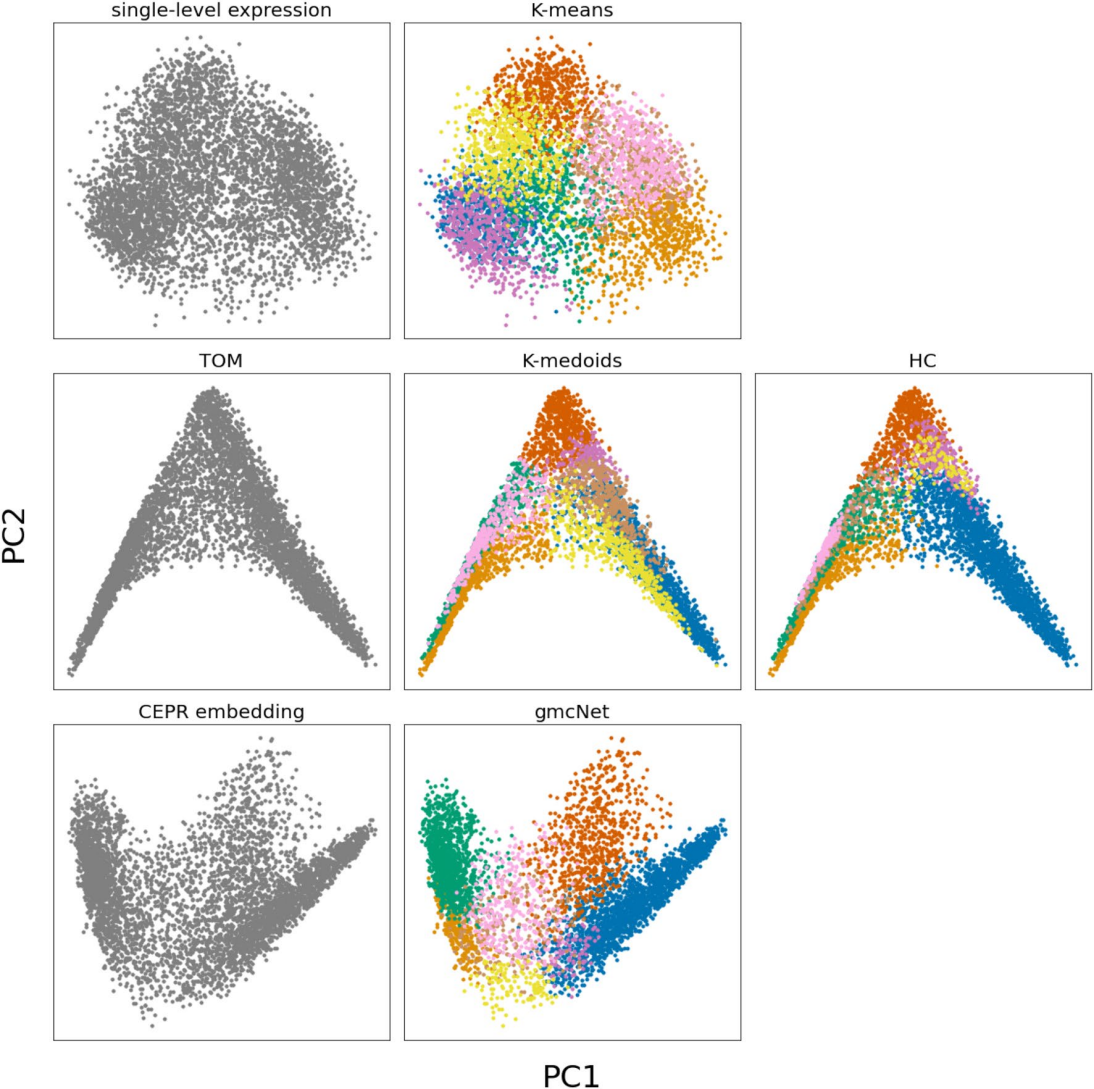
First and second principal components of three feature type of Korean native cattle dataset and clustering results of each method. The x-axis and y-axis are first and second principal component. The colors show the module memberships determined by the methods on the top.

Figure 2 shows plots based on the first and second principal components of three feature types (single-level expression, TOM, and CEPR embedding) of Korean native cattle dataset. Single-level expression fails to distinguish modules with ambiguous boundaries. This may reflect the low modularity of K-means, which uses single-level expression for clustering. The TOM provides stronger connections between genes than single-level expression. However, it also decreases the distances between different modules and genes. As shown in Fig. 2, K-medoids and HC, which use the TOM for clustering, do not clearly assign genes into different but closely related modules. Compared to the other types, CEPR embedding provides better separation, *i.e. *smaller distances between genes and larger ones between modules. With CEPR embedding, gmcNet defines gene modules more clearly and increases modularity.

**Figure 3:**
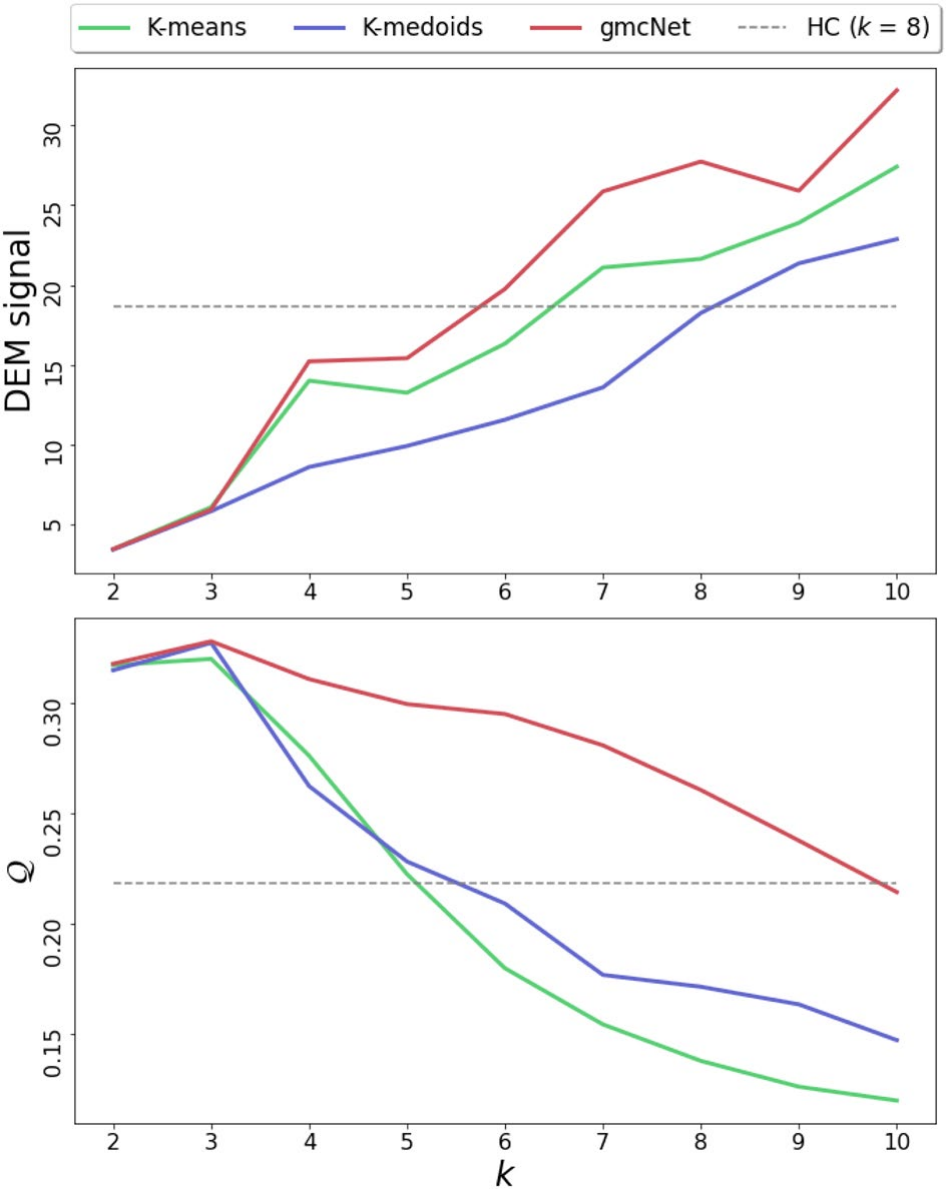
Optimal *k* searching considering DEM signaling and the modularity $\mathcal {Q}$.

**Figure 4:**
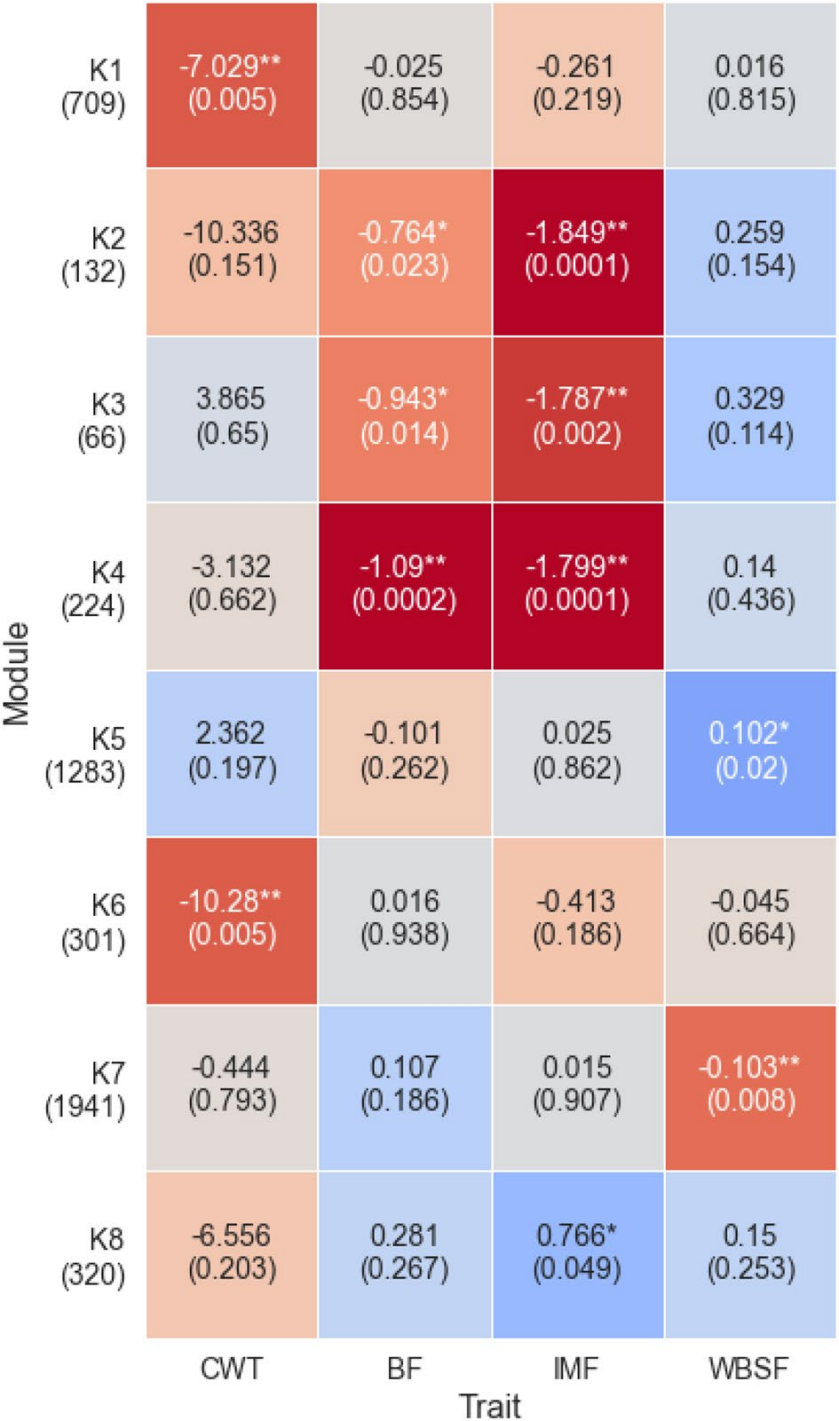
The DEM signals of modules defined by gmcNet. The y-axis shows the module names and numbers of genes within each module. The x-axis shows the complex traits. The numbers in each cell are regression coefficients (no parentheses) and the regression $p\text {-values}$ (in parentheses). Red and blue indicate negative and positive coefficients, respectively. *$p<0.05$, **$p<0.01$.

### Model performance at different *k* (number of clusters)

Our current implementation of gmcNet requires the setting of an optimal *k* (number of clusters). The effects of the *k*-value on modularity $\mathcal {Q}$ and the DEM signal are summarized in Fig. 3. With an increasing *k*-value, the DEM signal increases while the $\mathcal {Q}$ decreases. In contrast, gmcNet yields a larger DEM signal than HC even at smaller *k*-values ($6 \le k<8$), and remains higher $\mathcal {Q}$ at larger *k*-values ($k=9$). gmcNet outperforms K-means and K-medoids for all *k*-values. These results can demonstrate the superiority of gmcNet regardless of the *k*-value.

### Functional enrichment analysis of native Korean cattle

To identify the DEMs, we performed linear regression analysis of the module eigengenes^1^ for four complex traits, including carcass weight (CWT), backfat thickness (BF), intramuscular fat content (IMF), and the Warner-Bratzler shear force (WBSF). Figure 4 shows the results. In terms of the number of DEMs, IMF ranked first with four modules (K2, K3, K4, and K8) followed by BF (K2, K3, and K4), WBSF (K5 and K7), and CWT (K1 and K6). Interestingly, K5 and K7, which contain large numbers of genes, were significant to WBSF. This may reflect our mode of data collection; the RNA-seq data were from the *longissimus-dorsi* muscle and WBSF indicates the tenderness of beef muscle. Also, gmcNet detected 11 significant module-trait interactions. gmcNet found more DEMs than the other methods (HC: 9, K-means: 10, and K-medoids: 10) (Fig. S1).

**Figure 5:**
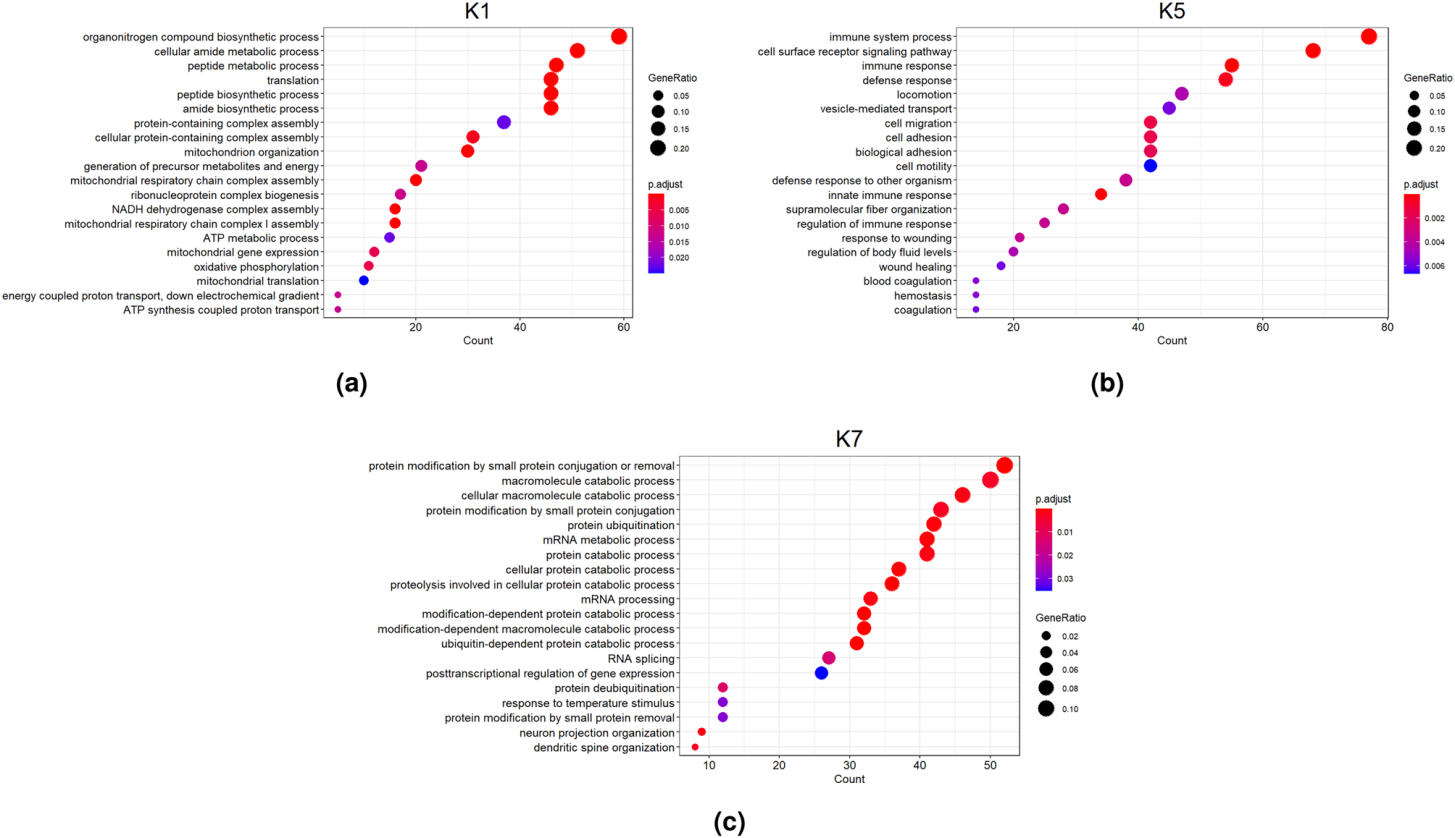
The biological processes of three significant modules: (**a**) K1, (**b**) K5, and (**c**) K7. p.adjust is a *p*-value adjusted by the Bonferroni method.

We used Gene Ontology (GO) enrichment analysis^21^ to annotate the biological processes of the modules defined by gmcNet. Three modules (K1, K5, and K7) were linked to significant processes (Fig. 5). K1, a CWT-related module, was enriched in “biosynthetic” and “metabolic” processes. Based on both the DEM analysis and the GO enrichment results, K1 seems to involve many genes associated with growth-related traits. Two WBSF-related modules (K5 and K7) were enriched in “immune system” and “protein catabolism” , respectively. Although several studies have suggested that the immune system plays a key role in cattle weight gain and feed efficiency^22,23^, the association between beef tenderness and immune pathways is a novel finding. Various studies have reported an association between “protein catabolic process” and beef tenderness^24–26^. Therefore, the results suggest that K7 is a key module of beef tenderness in native Korean cattle.

### Hub gene searches for modules of interest

**Figure 6:**
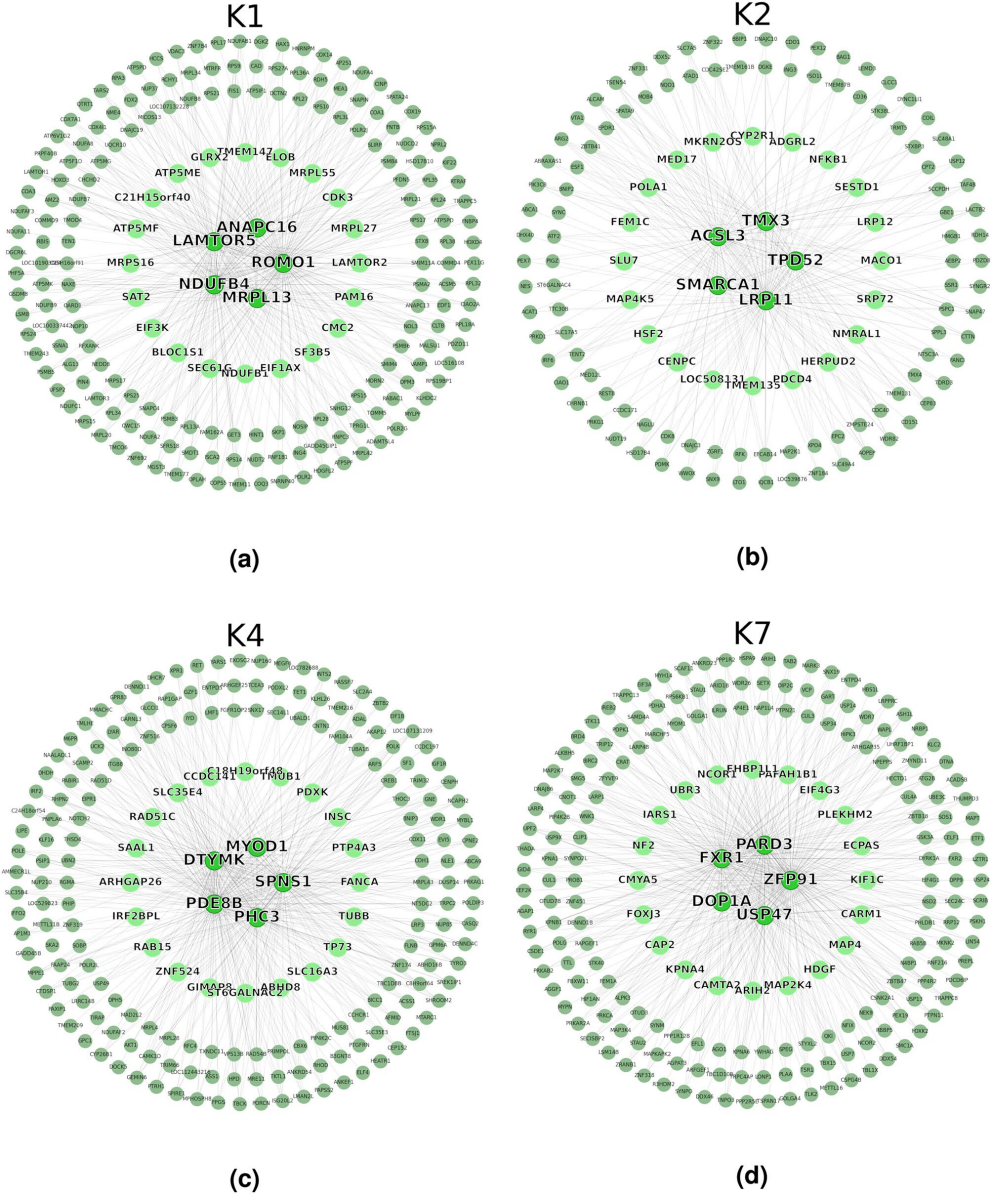
Hub gene networks of the four principal modules of native Korean cattle: (**a**) K1, (**b**) K2, (**c**) K4, (**d**) K7. From the outside in, the top 200, top 25, and top 5 hub genes are shown. The linkages of the top 5 hub genes are shown as the edges of the networks.

Given the functional enrichment results, we selected the four modules, K1, K2, K4, and K7, as the principal modules of complex traits. Figure 6 shows the hub gene networks and Table 2 shows the related traits. The six hub genes of K1 are related to quantitative traits including growth (*LAMTOR5*^27^ and *PAM16*^28^) and feed intake (*NDUFB1*^29^, *NDUFB4*^30^, *ATP5MF*^31^, and *SEC61G*^32^). These findings support our suggestion that K1 is significant in terms of CWT. K2 and K4, associated with fat-related traits (BF and IMF) in DEM analysis, include eight (*ACSL3*^33^, *NFKB1*^34^, *CYP2R1*^35^, *HSF2*^36^, *TMEM135*^37^, *PDCD4*^38^, *HERPUD2*^39^, and *NMRAL1*^40^) and seven (*SPNS1*^34^, *MYOD1*^41^, *PDXK*^42^, *TMUB1*^37^, *ARHGAP26*^43^, *RAB15*^34^, and *TP73*^44^) fat-related hub genes, respectively. Thus, future research should identify the relationships between fat metabolism and modules K2 and K4. Although K7 was associated with WBSF in DEM analysis, only four hub genes (*PARD3*^45^, *EIF4G3*^46^, *PAFAH1B1*^47^, and *CAMTA2*^48^) were associated with growth-related traits; the other hub genes were all novel.

**Table 2:** Hub genes and associated traits of the main modules.

Module	Hub gene$^1$	Significant trait$^2$	Reported cattle traits affected
K1	*ROMO1*, *ANAPC16*, *LAMTOR5*, *NDUFB4*, *MRPL13*, *LAMTOR2*, *MRPL27*, *CDK3*, *MRPL55*, *ELOB*, *TMEM147*, *GLRX2*, *ATP5ME*, *C21H15orf40*, *ATP5MF*, *MRPS16*, *SAT2*, *EIF3K*, *BLOC1S1*, *SEC61G*, *NDUFB1*, *EIF1AX*, *SF3B5*, *CMC2*, *PAM16*	CWT** (0.005)	Growth^27,28^; Tenderness^7,49,50,50^; Feed intake^29,31,32^; Fat^34,51,52^
K2	*TPD52*, *TMX3*, *ACSL3*, *SMARCA1*, *LRP11*, *MACO1*, *LRP12*, *SESTD1*, *NFKB1*, *ADGRL2*, *CYP2R1*, *MKRN2OS*, *MED17*, *POLA1*, *FEM1C*, *SLU7*, *MAP4K5*, *HSF2*, *CENPC*, *LOC508131*, *TMEM135*, *PDCD4*, *HERPUD2*, *NMRAL1*, *SRP72*	BF* (0.023); IMF** (0.0001)	Fat^33–35^; Growth^53–55^
K4	*SPNS1*, *MYOD1*, *DTYMK*, *PDE8B*, *PHC3*, *FANCA*, *PTP4A3*, *INSC*, *PDXK*, *TMUB1*, *C18H19orf48*, *CCDC141*, *SLC35E4*, *RAD51C*, *SAAL1*, *ARHGAP26*, *IRF2BPL*, *RAB15*, *ZNF524*, *GIMAP8*, *ST6GALNAC2*, *ABHD8*, *SLC16A3*, *TP73*, *TUBB*	BF** (0.0002); IMF** (0.0001)	Fat^34,41,42^; Growth^51,56,57^; Feed intake^5,58,59^; Tenderness^60,61^
K7	*ZFP91*, *PARD3*, *FXR1*, *DOP1A*, *USP47*, *KIF1C*, *ECPAS*, *PLEKHM2*, *EIF4G3*, *PAFAH1B1*, *EHBP1L1*, *NCOR1*, *UBR3*, *IARS1*, *NF2*, *CMYA5*, *FOXJ3*, *CAP2*, *KPNA4*, *CAMTA2*, *ARIH2*, *MAP2K4*, *HDGF*, *MAP4*, *CARM1*	WBSF** (0.008)	Fat^62^; Growth^45–47^

Hub gene$^1$: Top 25 hub genes; Significant trait$^2$: Significant traits revealed by DEM analysis (*p*-values). The reported traits affected by each hub gene are listed in S4 Table.

### Gene Expression Omnibus (GEO) repository

We also performed our method on the NCBI GEO datasets^19^. The datasets include four different species (GDS6010: human, GDS5618: mouse, GDS4246: pig, and GDS3857: chicken). We measured DEM signals using the trait included in each dataset (human: virus infection, mouse: pancreatic islets, pig: blood, chicken: light pulse). The implementation details for GEO datasets can be shown in supproting information S2. Table 3 presents the performances of the various methods on GED datasets. For mouse and chicken gmcNet achieves the best cluster modularity, while for human and pig gmcNet show much lower modularity than other TOM-based method (HC and K-medoids). However, gmcNet outperforms all methods on DEM signal capture with reasonable modularity for all datasets. These results can prove the gmcNet is useful method to group thousands of gene according to their system-level functionality.

**Table 3:** Model performance on GEO dataset in terms of graph modularity $\mathscr{Q}$ and DEM signaling.

	Method	HC	K-means	K-medoids	gmcNet
Human	$\mathscr{Q}$	0.255	0.155	**0.276**	0.231
	DEM-signal	21.620	24.975	22.082	**27.558**
Mouse	$\mathscr{Q}$	0.174	0.088	0.146	**0.186**
	DEM-signal	66.505	74.591	65.494	**76.680**
Pig	$\mathscr{Q}$	**0.181**	0.122	0.177	0.132
	DEM-signal	14.11	20.388	14.182	**21.295**
Chicken	$\mathscr{Q}$	0.328	0.334	0.265	**0.366**
	DEM-signal	25.95	31.709	23.65	**33.083**

Significant values are in [bold].

## Discussion

Single-level expression is generally appropriate to identify trait-specific marker genes that are differentially expressed depending on the biological phenotype^63^. Here, we found that single-level expression also revealed trait-specific modules with strong DEM signals. However, most existing WGCNA methods address only the co-expression topology (including TOM); the DEM signals are weak. On the other hand, our gmcNet simultaneously addresses single-level expression and TOM. gmcNet thus yielded larger DEM signals than other clustering methods. Furthermore, gmcNet produced some novel and interesting results. Threfore, gmcNet can detect module functionality and improves our understanding of WGCNA system-level biology. Also, gmcNet yields strong adjacencies between genes in the same module. gmcNet exploits the learnable properties of CEPR, which aggregates single-gene expressions with the co-expression features of its first neighbors, embedding these features to reduced dimensions. As noted in the Results section, CEPR generates more robust features than single-gene expression data or TOM. Given the CEPR-embedded feature, gmcNet achieved the best WGCNA modularity of all clustering methods tested.

Many genes are uniformly expressed in all individuals. Such genes (“noise”) are intimately connected with nested modules and exhibit no differential expression in complex trait analysis. Any attempt to cluster them disrupts module identification and obscures the biological implications. HC uses a dendrogram cut-off to exclude noisy genes. On the other hand, gmcNet assigns every gene to the most probable module. This may yield some meaningless assignments, because uniform expression may render the assignments to nested modules similar. Therefore, in future, it will be important to eliminate noise. We are exploring probability thresholding to this end. Specifically, genes with maximum probabilities lower than a given threshold will be excluded from module assignment. We will also add the optimal *k* search method to gmcNet; *k*-values can greatly increase model performance and may be modified depending on the characteristics of a dataset. Here, gmcNet used the optimal *k* of HC and performed better than other methods. In addition, gmcNet outperformed K-means and K-medoids at all *k*-values tested (2-10). Thus, the addition of an optimal *k* search would improve gmcNet performance in the context of WGCNA.

We derived a gene module clustering network, gmcNet, which simultaneously addresses single-level expression and TOM. We validated gmcNet performance using 4,976 genes from 20 native Korean cattle and four GEO datasets. gmcNet reliably assigned genes to modules exhibiting high modularity and DEM signals. gmcNet also detected some interesting biological functionalities. Therefore, gmcNet is a useful framework for WGCNA module clustering.

## Materials and methods

### Korean native cattle data

A total of 20 native Korean steers, born 2013 at Hanwoo Experiment Station, National Institute of Animal Science (NIAS), Rural Development Administration, South Korea, were used; all were humanely slaughtered at 30 months of age. The CWT (kg), and BF (mm) were measured after chilling for 24 hours. BF was measured at the junction of the 12th and 13th ribs. The WBSF and IMF were measured at the *longissimus-dorsi* muscle according to^64^ and^65^, respectively. RNA from the *longissimus-dorsi* muscle was extracted using TRIzol reagent (Invitrogen, Carlsbad, CA, USA). RNA quality and quantity were assessed by automated capillary gel electrophoresis performed using a Bioanalyzer 2100 running the RNA 6000 Nano LabChip (Agilent Technologies Ireland, Dublin, Ireland). Only RNA samples with RNA integrity $\ge 7$ were retained. Complementary DNA (cDNA) libraries were synthesized with Illumina TruSeq preparation Kit according to the manufacturer’s instruction (Illumina, San Diego, CA, USA). The RNA sequencing was done using Hiseq 2000 Illumina platform to obtain paired-end reads. The quality of the raw RNA samples was confirmed using FastQC v0.11^66^, and the reads with low quality were removed using Trimmomatic v0.36^67^. The reads were aligned to the reference genome Bos taurus (Ensemble UMD3.1) with TopHat v2.1^68^. The gene count of the reads was done with HTSeq v0.91^69^. Reads per kilobase per million (RPKM) were computed for each gene. We used Pearson correlation test to filter out uniformly expressed genes for the four traits (CWT, BF, IMF, and WBSF). Specifically, we calculated correlation coefficients between each gene and the traits. Then, the genes which show non-significant correlation ($p\text {-value}>0.1$) for any of the traits, were excluded in further progresses. After deriving Pearson correlation test, we excluded 7,555 genes and subjected 4,976 genes in 20 samples to this study. Notice that the National Institute of Animal Science (NIAS) of the Rural Development Administration (RDA) of South Korea approved the experimental procedures (ethics committee approval number: 2015-150).

### Co-expression network construction

To represent the co-expression network in matrix form, we used the topological overlap matrix of^1^. Briefly, the adjacency of each pair of genes *i* and *j* is given by $a_{ij}=\left| {cor}_{ij}\right| ^{\beta }$ where $\beta$ is a smoothing parameter and ${cor}_{ij}$ is the correlation coefficient between the single-level expressions of the two genes. Given the adjacency values $a_{ij}$, the topological overlap matrix $\mathbf {T}\in \mathbb {R}^{n\times n}$ was created using a TOM^70^, where *n* is the number of genes. TOM $t_{ij}$, which provides a similarity measure in the topological overlap matrix, is calculated as follows:

1 $$\begin{aligned} t_{ij} = \frac{l_{ij} + a_{ij}}{\text {min}\{k_i,k_j\}+1-a_{ij}} \end{aligned}$$

where, $l_{ij}=\sum _u{a_{iu}a_{uj}}$ and $k_i=\sum _u{a_{iu}}$ is a node connectivity.

**Figure 7:**
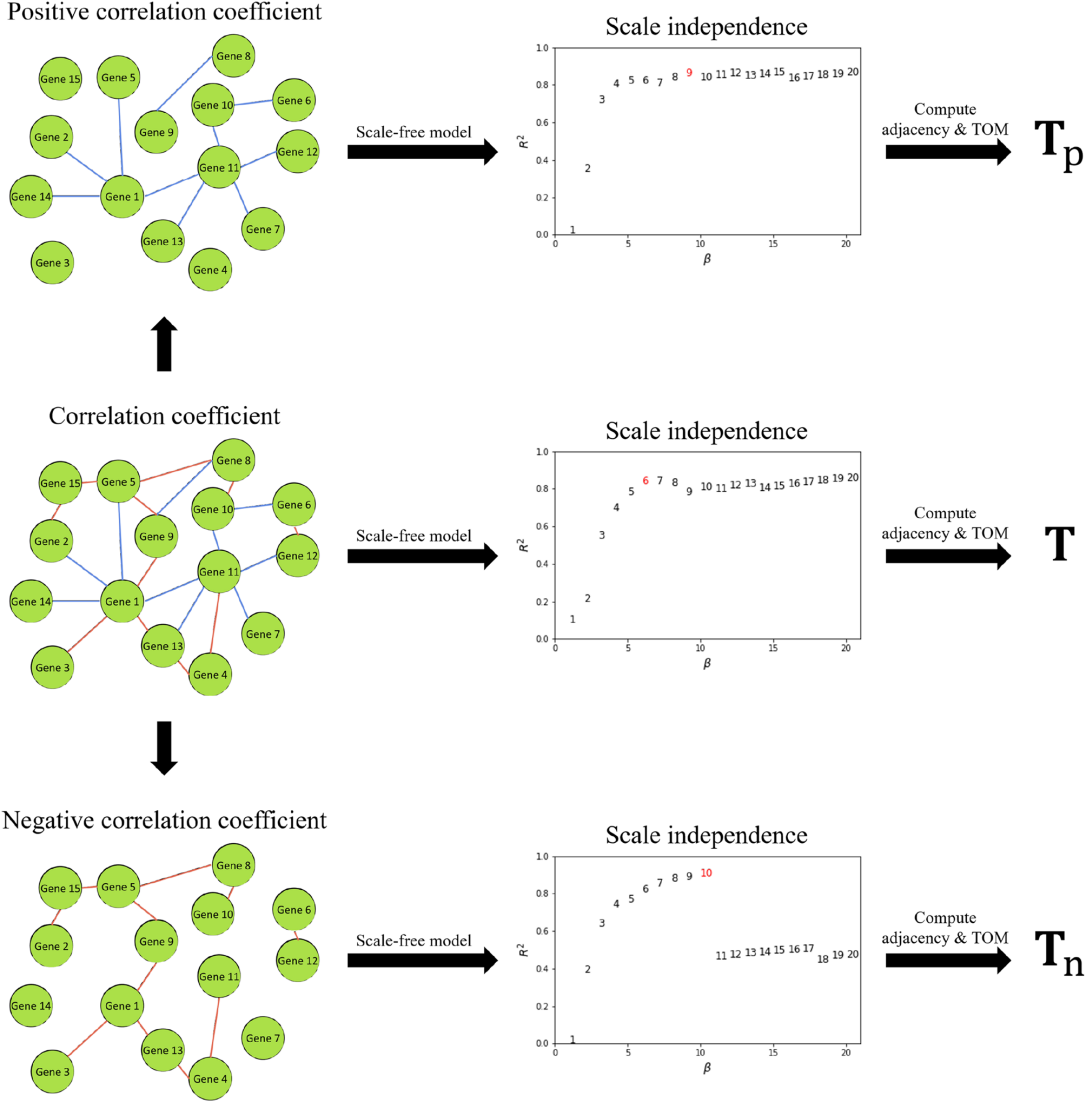
Construction of three topological overlap matrices. $\mathbf {T}$ is the topological overlap matrix of all relationships. $\mathbf {T}_\text {p}$ and $\mathbf {T}_\text {n}$ are the topological overlap matrices of positive and negative relationships respectively.

Also, we constructed two additional topological overlap matrices to train gmcNet (Fig. 7). $\mathbf {T}_\text {p}\in \mathbb {R}^{n\times }$, representing the positive network, was created leaving only positive correlation coefficients, whereas $\mathbf {T}_\text {n}\in \mathbb {R}^{n\times n}$, representing the negative network, was created leaving only negative correlation coefficients. After scale-free model fitting^1^, we chose $\beta =6$, $\beta =9$, and $\beta =10$ as the smoothing parameters for $\mathbf {T}$, $\mathbf {T}_\text {p}$, and $\mathbf {T}_\text {n}$, respectively.

### Gene module clustering network

**Figure 8:**
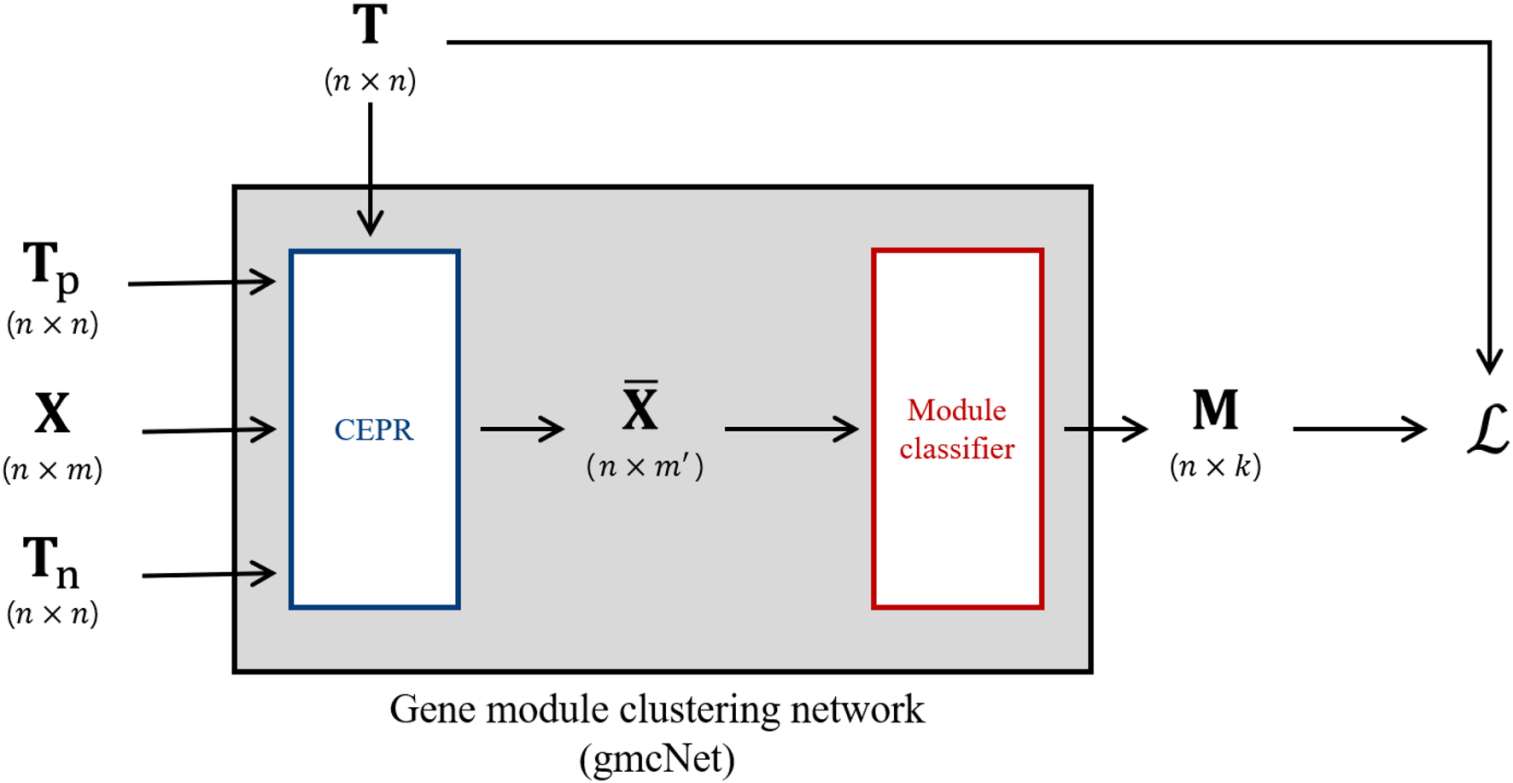
The architecture of gmcNet. $\mathbf {X}\in \mathbb {R}^{n\times m}$ is the single-level expression of *n* genes in *m* samples. $\bar{\mathbf {X}}\in \mathbb {R}^{n\times m^\prime }$ is CEPR-embedded feature with $m^\prime$ dimension. $\mathbf {M}\in \mathbb {R}^{n\times k}$ is assignment probability matrix of *n* genes to *k* modules. $\mathscr{L}$ is loss function.

We developed a gene module clustering network (gmcNet) that clusters genes according to their co-expression topologies (genes in the same module should be strongly connected) and their single-level expression (genes in the same module should exhibit similar expression patterns). Figure 8 shows an overview of gmcNet, which features a co-expression pattern recognizer (CEPR) and module classifier. The CEPR incorporates the expression features of single genes into the topological features of co-expressed ones. Given this CEPR-embedded feature, the module classifier computes module assignment probabilities.

#### Network structure

**CEPR:** The goal of CEPR is to integrate single-expression features with co-expression features. To achieve this, we used the MP operation of MPNN^11^, but employed the topological overlap matrix rather than the adjacency matrix. We computed a new topological overlap matrix $\widetilde{\mathbf {T}}$ by zeroing the diagonal of $\mathbf {T}$ and applying degree normalization:

2 $$\begin{aligned} \begin{array}{ll} \mathbf {T}_\text {z}=\mathbf {T}-\mathbf {I}_n;&\widetilde{\mathbf {T}}=\mathbf {D}^{-\frac{1}{2}}\mathbf {T}_\text {z}\mathbf {D}^{-\frac{1}{2}} \end{array} \end{aligned}$$

where $\mathbf {D}=\text {diag}({\mathbf {T}_\text {z}1}_n)$ is a degree matrix. Let $\mathbf {X}\in \mathbb {R}^{n\times m}$ be the single-level expression of *n* genes in *m* samples. Then, single and co-expression can be simply combined via an MP operation:

3 $$\begin{aligned} \mathrm {MP}(\mathbf {X},\widetilde{\mathbf {T}})=\text {ReLU}(\widetilde{\mathbf {T}}\mathbf {X}\mathbf {W}_{\text {co}}+\mathbf {X}\mathbf {W}_{\text {single}}) \end{aligned}$$

where $\mathbf {W}_\text {co}$ and $\mathbf {W}_\text {single}$ are the trainable parameters of the co- and single-expression features. As $\widetilde{\mathbf {T}}$ includes the topological adjacencies between gene pairs, it is easy to see that $\widetilde{\mathbf {T}}\mathbf {X}$ can be interpreted as a co-expression feature.

A simple MP operation cannot separate positive and negative co-expressions, even when they differ in different biological pathways. Therefore, we refined a simple MP to become a CEPR, as follows:

4 $$\begin{aligned} \bar{\mathbf {X}}= \mathrm {CEPR}(\mathbf {X},\widetilde{\mathbf {T}},{\widetilde{\mathbf {T}}}_\text {p},{\widetilde{\mathbf {T}}}_\text {n})= \text {ReLU}(\widetilde{\mathbf {T}}\mathbf {X}\mathbf {W}_\text {c}+ \widetilde{\mathbf {T}}_\text {p}\mathbf {X}\mathbf {W}_\text {p}+ {\widetilde{\mathbf {T}}}_\text {n}\mathbf {X}\mathbf {W}_\text {n}+ \mathbf {X}\mathbf {W}_\text {s}) \end{aligned}$$

where $\{\mathbf {W}_\text {c}, \mathbf {W}_\text {p}, \mathbf {W}_\text {n}, \mathbf {W}_\text {s}\} \in \mathbb {R}^{m\times m^\prime }$ are the trainable weights of the co-expression, positive co-expression, negative co-expressions, and single-expression, respectively. $m^\prime$ is an embedding dimension (set to 8). As ${\widetilde{\mathbf {T}}}_\text {p}\mathbf {X}\mathbf {W}_\text {p}$ and ${\widetilde{\mathbf {T}}}_\text {n}\mathbf {X}\mathbf {W}_\text {n}$ are identical in terms of dimensionality, CEPR learns various co-expressions by simply adding them. By skip connections of single-expression $\mathbf {X}\mathbf {W}_\text {s}$, CEPR generates the embedding feature $\bar{\mathbf {X}}\in \mathbb {R}^{n\times m^\prime }$, which deals with single-expression and three different co-expressions in the $m^\prime$ dimension.

**Module classifier:** Given the CEPR-embedded feature $\bar{\mathbf {X}}$, the module classifier computes a module assignment probability using a multi-layer perceptron (MLP):

5 $$\begin{aligned} \mathbf {M}=\text {softmax}(\bar{\mathbf {X}}\mathbf {W}_\text {m}) \end{aligned}$$

where $\mathbf {W}_\text {m}\in \mathbb {R}^{m^\prime \times k}$ are the trainable weights for clustering of *k* modules. As softmax activation guarantees that $m_{ij}\in [0,1]$, the *i*th-row of $\mathbf {M}\in \mathbb {R}^{n\times k}$ corresponds to the module-assignment probability of gene *i*. In other words, gene *i* belongs to module *c* if $m_{ic}$ is the maximum value of the *i*th-row of $\mathbf {M}$.

#### Loss function

For unsupervised clustering, we employed the cut and orthogonality loss terms of MinCutPool^71^. The loss function when training gmcNet was defined as:

6 $$\begin{aligned} \mathscr{L}= {\lambda \mathscr{L}}_\text {c}+\mathscr{L}_\text {o}= \lambda \underbrace{\left( -\frac{Tr(\mathbf {M}^\text {T}\widetilde{\mathbf {T}}\mathbf {M})}{Tr(\mathbf {M}^\text {T}\widetilde{\mathbf {D}}\mathbf {M})}\right) }_{\mathscr{L}_\text {c}}+ \underbrace{\left\| {\frac{\mathbf {M}^\text {T}\mathbf {M}}{{\left\| \mathbf {M}^\text {T}\mathbf {M} \right\| }_F} + \frac{\mathbf {I}_k}{\sqrt{k}}}\right\| _F}_{\mathscr{L}_\text {o}} \end{aligned}$$

where ${\left\| \cdot \right\| }_F$ indicates the Frobenius norm and *Tr* is the trace; $\lambda$ is a balancing hyper-parameter, which is set to 2.6. The cut loss term, $\mathscr{L}_\text {c}$, encourages clustering of strongly connected genes within the same module, and the orthogonality loss term, $\mathscr{L}_\text {o}$, penalizes assignment to similarly sized modules.

#### Implementation Details

The model was iterated for 5,000 epochs using a GeForce RTX 2080ti. For the first 100 epochs, the balancing hyperparameter $\lambda$ was set to 0 and the learning rate to 0.01. This prevented the creation of empty modules. After epoch 100, we set $\lambda$ to 2.6 and the learning rate to 0.001. Model training was early stopped at $\mathscr{L}_\text {o}>\tau$, where $\tau$ is the orthogonal threshold, which was set to 0.8. The Adam optimizer^72^ was used to minimize the loss function. Finally, $\mathbf {M}$ at the end of training was used for module assignment.

### Model performance

To validate gmcNet performance, HC^7^, K-means^73^ and K-medoids^74^ were also used for module clustering and the results were compared to those of gmcNet. K-means uses single-expression feature $\mathbf {X}$ as input data; the HC and K-medoids use the topological distances $1-\mathbf {T}$ as inputs. The optimal *k* for K-means, K-medoids, and gmcNet was set to 8, as suggested by application of the dynamic tree cut technique^7^ to HC.

#### Metrics

We measured the model performance in terms of modularity and DEM signaling. Module modularity is a commonly used metric in graph clustering. In a fully random graph, gene *i* and *j* of degrees $c_i=\sum _{u}t_{iu}$ and $c_j=\sum _{u}t_{ju}$ are connected with a probability $c_ic_j/s$, where *s* is the total topological overlap $s=\sum _{ij}{t_{ij}}$. Modularity measures the divergence between intra-module connections as:

7 $$\begin{aligned} \mathscr{Q}=\frac{1}{s}\sum _{ij}^{n}{(t_{ij}-\frac{c_ic_j}{s}})\delta \left[ i,j\right] \end{aligned}$$

where $\delta \left[ i,j\right] =1$ if *i* and *j* belong to the same module; otherwise, $\delta \left[ i,j\right] =\ 0$.

To assess functional enrichment of clustering method, we introduce a novel metric, called DEM signal. Let $\rho [l,t]=1$ if module *l* is significant ($\le 0.05$) for trait *t*; otherwise, $\rho \left[ l,t\right] =0$. The final DEM signal was defined as:

8 $$\begin{aligned} \text {DEM signal}=\sum _{l}^{k}\sum _{t}-\text {log}_{10}(p\text {-value}_{lt})\rho [l,t] \end{aligned}$$

where *t* is traits and $p\text {-value}_{lt}$ indicates the significance value of module *l* in terms of trait *t*. We employed linear regression analysis to the module eigengenes, *i.e. *the first principal components of the modules, for four complex traits: CWT, BF, IMF and WBSF.

#### Functional enrichment analysis

The Bioconductor R package “clusterProfiler”^75^ was used for GO analysis. The adjusted $p\text {-value}$ (obtained using the Bonferroni method) was employed to examine the significance (p.adjust$<0.05$) of all GO terms. The top 20 biological processes were extracted if there were more than 20 significant results. To identify hub genes, we calculated the correlation coefficients between single-level expression of each gene and the ME of the module it belong to. The top 25 genes (in terms of correlation coefficients) were defined as hub genes.

## Supplementary material

Below is the link to the electronic supplementary material.

Supplementary Information 1.

Supplementary Information 2.

Supplementary Information 3.

Supplementary Information 4.

## Data Availability

The gmcNet code and example data is available on GitHub at https://github.com/gywns6287/gmcNet. Request for full gene expression data of Korean native cattle can be made to Korea National Institute of Animal Science, Animal Genome & Bioinformatics Division (http://www.nias.go.kr/english/sub/boardHtml.do?boardId=depintro).
