# Supplementary material for: Use of a graph neural network to the weighted gene co-expression network analysis of Korean native cattle
Source: Sci Rep. 2022 Jun 14;12:9854. doi: 10.1038/s41598-022-13796-9 (PMC9197844; doi:10.1038/s41598-022-13796-9)
Supplement: Supplementary file 1 — Supplementary Information 1. [file 41598_2022_13796_MOESM1_ESM.pdf]

Reported trait for hub genes.

Module	Reported trait	Gene
K1	Fertility	*ROMO1* [35]
	Fertility	*C21H15orf40* [40]
	Fat	*ANAPC16* [11]
	Fat	*MRPL27* [46]
	Fat	*CDK3* [64]
	Growth	*LAMTOR5* [52]
	Growth	*PAM16* [25]
	Calf mortality	*LAMTOR5* [37]
	Calf mortality	*BLOC1S1* [37]
	Tenderness	*NDUFB4* [5]
	Tenderness	*ELOB* [43]
	Tenderness	*TMEM147* [28]
	Tenderness	*ATP5ME* [43]
	Heat stress	*MRPL55* [22]
	Feed intake	*NDUFB4* [4]
	Feed intake	*ATP5MF* [45]
	Feed intake	*SEC61G* [18]
	Feed intake	*NDUFB1* [24]
	Milk	*PAM16* [20]
K2	Fat	*ACSL3* [34]
	Fat	*NFKB1* [64]
	Fat	*CYP2R1* [31]
	Fat	*HSF2* [2]
	Fat	*TMEM135* [55]
	Fat	*PDCD4* [39]
	Fat	*HERPUD2* [69]
	Fat	*NMRAL1* [13]
	Fertility	*TPD52* [60]
	Fertility	*SMARCA1* [63]
	Fertility	*SESTD1* [57]
	Fertility	*POLA1* [61]
	Fertility	*HSF2* [14]
	Fertility	*PDCD4* [16]
	Fertility	*CENPC* [21]
	Growth	*TMX3* [9]

	Growth	*ACSL3* [1]
	Growth	*LRP12* [54]
	Growth	*SESTD1* [66]
	Growth	*PDCD4* [18]
	Growth	*NMRAL1* [25]
	Milk	*TPD52* [21]
	Milk	*ACSL3* [30]
	Milk	*LRP12* [21]
	Milk	*CENPC* [21]
	Feed intake	*CYP2R1* [67]
	Fat	*SPNS1* [64]
	Fat	*MYOD1* [56]
	Fat	*PDXK* [48]
	Fat	*TMUB1* [55]
	Fat	*ARHGAP26* [38]
	Fat	*RAB15* [64]
	Fat	*TP73* [10]
	Growth	*MYOD1* [11]
	Growth	*DTYMK* [36]
	Growth	*PDXK* [23]
	Growth	*ARHGAP26* [33]
	Growth	*RAD51C* [27]
	Growth	*IRF2BPL* [12]
K4	Fertility	*MYOD1* [47]
	Fertility	*PDE8B* [15]
	Fertility	*FANCA* [41]
	Fertility	*CCDC141* [51]
	Fertility	*RAD51C* [59]
	Fertility	*SLC16A3* [42]
	Feed intake	*GIMAP8* [53]
	Feed intake	*SLC16A3* [12]
	Feed intake	*TUBB* [26]
	Tenderness	*MYOD1* [62]
	Tenderness	*PDE8B* [29]
	Milk	*CCDC141* [68]
	Milk	*TUBB* [21]
	Fertility	*ZFP91* [17]

K7

	Fertility	*PARD3* [49]
	Fertility	*FXR1* [59]
	Fertility	*USP47* [59]
	Fertility	*PLEKHM2* [32]
	Fertility	*EIF4G3* [19]
	Fertility	*PAFAH1B1* [16]
	Fertility	*NCOR1* [71]
	Fertility	*FOXJ3* [65]
	Fertility	*KPNA4* [7]
	Fertility	*MAP2K4* [44]
	Growth	*PARD3* [70]
	Growth	*EIF4G3* [24]
	Growth	*PAFAH1B1* [58]
	Growth	*CMYA5* [6]
	Growth	*CAMTA2* [3]
	Fat	*MAP2K4* [50]
	Meat color	*PARD3* [37]
	Milk	*PARD3* [8]

References

[1] I. Anton, B. Húth, I. Füller, G. Gábor, G. Holló, and A. Zsolnai. Effect of single-nucleotide polymorphisms on the breeding value of fertility and breeding value of beef in hungarian simmental cattle. *Acta Veterinaria Hungarica*, 66(2):215–225, 2018.

[2] M. Baik, T. Vu, M. Piao, and H. Kang. Association of dna methylation levels with tissue-specific expression of adipogenic and lipogenic genes in longissimus dorsi muscle of korean cattle. *Asian-Australasian journal of animal sciences*, 27(10):1493, 2014.

[3] J. Bazile, F. Jaffrezic, P. Dehais, M. Reichstadt, C. Klopp, D. Laloë, and M. Bonnet. Molecular signatures of muscle growth and composition deciphered by the meta-analysis of age-related public transcriptomics data. *Physiological Genomics*, 52(8):322–332, 2020.

[4] P. D. B. Benedeti, E. Detmann, H. Mantovani, S. Bonilha, N. Serão, D. Lopes, W. Silva, C. Newbold, and M. Duarte. Nellore bulls (bos taurus indicus) with high residual feed intake have increased the expression of genes involved in oxidative phosphorylation in rumen epithelium. *Animal Feed Science and Technology*, 235:77–86, 2018.

[5] C. Bernard, I. Cassar-Malek, M. Le Cunff, H. Dubroeucq, G. Renand, and J.-F. Hocquette. New indicators of beef sensory quality revealed by expression of specific genes. *Journal of Agricultural and Food Chemistry*, 55(13):5229–5237, 2007.

[6] M. Carvalho, F. Baldi, P. Alexandre, M. Santana, R. Ventura, R. Bueno, M. Bonin, F. Rezende, L. Coutinho, and J. Eler. Genomic regions and genes associated with carcass quality in nelore cattle. *Genetics and Molecular Research*, 18(1):1–15, 2019.

[7] Z. Cheng, C. F. Oguejiofor, T. Swangchan-Uthai, S. Carr, and D. C. Wathes. Relationships between circulating urea concentrations and endometrial function in postpartum dairy cows. *Animals*, 5(3):748–773, 2015.

[8] J. B. Cole, G. R. Wiggans, L. Ma, T. S. Sonstegard, T. J. Lawlor, B. A. Crooker, C. P. Van Tassell, J. Yang, S. Wang, and L. K. Matukumalli.

Genome-wide association analysis of thirty one production, health, reproduction and body conformation traits in contemporary us holstein cows. *BMC genomics*, 12(1):1–17, 2011.

[9] M. H. de Almeida Santana, G. A. O. Junior, A. S. M. Cesar, M. C. Freua, R. da Costa Gomes, S. d. L. e Silva, P. R. Leme, H. Fukumasu, M. E. Carvalho, and R. V. Ventura. Copy number variations and genome-wide associations reveal putative genes and metabolic pathways involved with the feed conversion ratio in beef cattle. *Journal of applied genetics*, 57(4):495–504, 2016.

[10] S. de Las Heras-Saldana, K. Y. Chung, H. Kim, D. Lim, C. Gondro, and J. H. van der Werf. Differential gene expression in longissimus dorsi muscle of hanwoo steers—new insight in genes involved in marbling development at younger ages. *Genes*, 11(11):1381, 2020.

[11] M. V. A. de Lemos, E. Peripolli, M. P. Berton, F. L. B. Feitosa, B. F. Olivieri, N. B. Stafuzza, R. L. Tonussi, S. Kluska, H. L. J. Chiaia, and L. Mueller. Association study between copy number variation and beef fatty acid profile of nellore cattle. *Journal of applied genetics*, 59(2):203–223, 2018.

[12] A. A. Elolimy, S. J. Moisá, K. M. Brennan, A. C. Smith, D. Graugnard, D. W. Shike, and J. J. Loor. Skeletal muscle and liver gene expression profiles in finishing steers supplemented with amaize. *Animal Science Journal*, 89(8):1107–1119, 2018.

[13] B. Engle, M. Masters, J. A. Boles, and J. Thomson. Gene expression and carcass traits are different between different quality grade groups in red-faced hereford steers. *Animals*, 11(7):1910, 2021.

[14] P. A. Fonseca, A. Suárez-Vega, and A. Cánovas. Weighted gene correlation network meta-analysis reveals functional candidate genes associated with high-and sub-fertile reproductive performance in beef cattle. *Genes*, 11(5):543, 2020.

[15] K. P. Gaddis, D. Null, and J. Cole. Explorations in genome-wide association studies and network analyses with dairy cattle fertility traits. *Journal of dairy science*, 99(8):6420–6435, 2016.

[16] J. M. Galliou, J. N. Kiser, K. F. Oliver, C. M. Seabury, J. G. Moraes, G. W. Burns, T. E. Spencer, J. Dalton, and H. L. Neibergs. Identification of loci and pathways associated with heifer conception rate in us holsteins. *Genes*, 11(7):767, 2020.

[17] N. Ghanem, D. Salilew-Wondim, M. Hoelker, K. Schellander, and D. Tesfaye. Transcriptome profile and association study revealed stat3 gene as a potential quality marker of bovine gametes. *Zygote*, 28(2):116–130, 2020.

[18] W. L. Hamilton, R. Ying, and J. Leskovec. Inductive representation learning on large graphs. In *Proceedings of the 31st International Conference on Neural Information Processing Systems*, pages 1025–1035.

[19] J. K. Höglund, B. Buitenhuis, B. Guldbrandtsen, M. S. Lund, and G. Sahana. Genome-wide association study for female fertility in nordic red cattle. *BMC genetics*, 16(1):1–11, 2015.

[20] T. Iso-Touru, G. Sahana, B. Guldbrandtsen, M. Lund, and J. Vilkki. Genome-wide association analysis of milk yield traits in nordic red cattle using imputed whole genome sequence variants. *BMC genetics*, 17(1):1–12, 2016.

[21] J. Jiang, L. Ma, D. Prakapenka, P. M. VanRaden, J. B. Cole, and Y. Da. A large-scale genome-wide association study in us holstein cattle. *Frontiers in genetics*, 10:412, 2019.

[22] N. Kapila, A. Sharma, A. Kishore, M. Sodhi, P. K. Tripathi, A. K. Mohanty, and M. Mukesh. Impact of heat stress on cellular and transcriptional adaptation of mammary epithelial cells in riverine buffalo (bubalus bubalis). *PloS one*, 11(9):e0157237, 2016.

[23] B. N. Keel, C. M. Zarek, J. W. Keele, L. A. Kuehn, W. M. Snelling, W. T. Oliver, H. C. Freetly, and A. K. Lindholm-Perry. Rna-seq meta-analysis identifies genes in skeletal muscle associated with gain and intake across a multi-season study of crossbred beef steers. *BMC genomics*, 19(1):1–11, 2018.

[24] K. Keogh, S. M. Waters, P. Cormican, A. K. Kelly, E. O'Shea, and D. A. Kenny. Effect of dietary restriction and subsequent re-alimentation

on the transcriptional profile of bovine ruminal epithelium. *PloS one*, 12(5):e0177852, 2017.

[25] R. J. Kern, A. K. Lindholm-Perry, H. C. Freetly, W. M. Snelling, J. W. Kern, J. W. Keele, J. R. Miles, A. P. Foote, W. T. Oliver, and L. A. Kuehn. Transcriptome differences in the rumen of beef steers with variation in feed intake and gain. *Gene*, 586(1):12–26, 2016.

[26] R. S. Kong, G. Liang, Y. Chen, and P. Stothard. Transcriptome profiling of the rumen epithelium of beef cattle differing in residual feed intake. *BMC genomics*, 17(1):1–16, 2016.

[27] A. Kour, S. M. Deb, N. Nayee, S. K. Niranjan, V. S. Raina, A. Mukherjee, I. D. Gupta, and C. S. Patil. Novel insights into genome-wide associations in bos indicus reveal genetic linkages between fertility and growth. *Animal Biotechnology*, pages 1–17, 2021.

[28] P. Langfelder, B. Zhang, and S. Horvath. Defining clusters from a hierarchical cluster tree: the dynamic tree cut package for r. *Bioinformatics*, 24(5):719–720, 2008.

[29] J. D. Leal-Gutiérrez, M. A. Elzo, D. D. Johnson, H. Hamblen, and R. G. Mateescu. Genome wide association and gene enrichment analysis reveal membrane anchoring and structural proteins associated with meat quality in beef. *BMC genomics*, 20(1):1–18, 2019.

[30] C. Li, D. Sun, S. Zhang, S. Wang, X. Wu, Q. Zhang, L. Liu, Y. Li, and L. Qiao. Genome wide association study identifies 20 novel promising genes associated with milk fatty acid traits in chinese holstein. *PloS one*, 9(5):e96186, 2014.

[31] Y. Li, M. Wang, Q. Li, Y. Gao, Q. Li, J. Li, and Y. Cao. Transcriptome profiling of longissimus lumborum in holstein bulls and steers with different beef qualities. *PloS one*, 15(6):e0235218, 2020.

[32] A. Liu, Y. Wang, G. Sahana, Q. Zhang, L. Liu, M. S. Lund, and G. Su. Genome-wide association studies for female fertility traits in chinese and nordic holsteins. *Scientific reports*, 7(1):1–12, 2017.

[33] M. Londoño-Gil, J. C. R. Flórez, A. Lopez-Herrera, and L. G. Gonzalez-Herrera. Genome-wide association study for growth traits in blanco

orejinero (bon) cattle from colombia. *Livestock Science*, 243:104366, 2021.

[34] Y. Lv, Y. Cao, Y. Gao, J. Yun, Y. Yu, L. Zhang, Z. Hu, L. Liu, J. Xue, and G. Zhang. Effect of acsl3 expression levels on preadipocyte differentiation in chinese red steppe cattle. *DNA and cell biology*, 38(9):945–954, 2019.

[35] Y. Lv, S. Ji, X. Chen, D. Xu, X. Luo, M. Cheng, Y. Zhang, X. Qu, and Y. Jin. Effects of crocin on frozen-thawed sperm apoptosis, protamine expression and membrane lipid oxidation in yanbian yellow cattle. *Reproduction in Domestic Animals*, 55(8):1011–1020, 2020.

[36] E. Manca, A. Cesarani, G. Gaspa, S. Sorbolini, N. P. Macciotta, and C. Dimauro. Use of the multivariate discriminant analysis for genome-wide association studies in cattle. *Animals*, 10(8):1300, 2020.

[37] N. Marín-Garzón, A. Magalhães, P. Schmidt, M. Serna, L. Fonseca, B. Salatta, G. Frezarim, G. Fernandes-Júnior, T. Bresolin, and R. Carvalheiro. Genome-wide scan reveals genomic regions and candidate genes underlying direct and maternal effects of preweaning calf mortality in nellore cattle. *Genomics*, 113(3):1386–1395, 2021.

[38] R. Martins, P. C. Machado, L. F. B. Pinto, M. R. Silva, F. S. Schenkel, L. F. Brito, and V. B. Pedrosa. Genome-wide association study and pathway analysis for fat deposition traits in nellore cattle raised in pasture–based systems. *Journal of Animal Breeding and Genetics*, 138(3):360–378, 2021.

[39] B. C. Melnik, S. M. John, and G. Schmitz. Milk consumption during pregnancy increases birth weight, a risk factor for the development of diseases of civilization. *Journal of Translational Medicine*, 13(1):1–11, 2015.

[40] A. Mohammadi, S. Alijani, S. A. Rafat, and R. Abdollahi-Arpanahi. Genome-wide association study and pathway analysis for female fertility traits in iranian holstein cattle. *Annals of Animal Science*, 20(3):825–851, 2020.

[41] S. G. Moore, J. E. Pryce, B. J. Hayes, A. J. Chamberlain, K. E. Kemper, D. P. Berry, M. McCabe, P. Cormican, P. Lonergan, and T. Fair. Differentially expressed genes in endometrium and corpus luteum of holstein cows selected for high and low fertility are enriched for sequence variants associated with fertility. *Biology of reproduction*, 94(1):19, 1–11, 2016.

[42] S. E. Moorey, J. M. Monnig, M. F. Smith, M. S. Ortega, J. A. Green, K. G. Pohler, G. A. Bridges, S. K. Behura, and T. W. Geary. Differential transcript profiles in cumulus-oocyte complexes originating from pre-ovulatory follicles of varied physiological maturity in beef cows. *Genes*, 12(6):893, 2021.

[43] M. M. M. Muniz, L. F. S. Fonseca, D. B. dos Santos Silva, H. R. de Oliveira, F. Baldi, A. L. Chardulo, J. A. Ferro, A. Cánovas, and L. G. de Albuquerque. Identification of novel mrna isoforms associated with meat tenderness using rna sequencing data in beef cattle. *Meat Science*, page 108378, 2020.

[44] M. Neupane, T. W. Geary, J. N. Kiser, G. W. Burns, P. J. Hansen, T. E. Spencer, and H. L. Neibergs. Loci and pathways associated with uterine capacity for pregnancy and fertility in beef cattle. *PLoS One*, 12(12):e0188997, 2017.

[45] W. Nolte, R. Weikard, R. M. Brunner, E. Albrecht, H. M. Hammon, A. Reverter, and C. Kühn. Identification and annotation of potential function of regulatory antisense long non-coding rnas related to feed efficiency in bos taurus bulls. *International journal of molecular sciences*, 21(9):3292, 2020.

[46] B. F. Olivieri, C. U. Braz, F. Brito Lopes, E. Peripolli, R. Medeiros de Oliveira Silva, R. Ruegger Pereira da Silva Corte, L. G. d. Albuquerque, A. S. C. Pereira, N. B. Stafuzza, and F. Baldi. Differentially expressed genes identified through rna-seq with extreme values of principal components for beef fatty acid in nelore cattle. *Journal of Animal Breeding and Genetics*, 138(1):80–90, 2021.

[47] F. Paradis, K. M. Wood, K. C. Swanson, S. P. Miller, B. W. McBride, and C. Fitzsimmons. Maternal nutrient restriction in mid-to-late gestation influences fetal mrna expression in muscle tissues in beef cattle. *BMC genomics*, 18(1):1–14, 2017.

[48] S. Peletto, M. Strillacci, M. Capucchio, E. Biasibetti, P. Modesto, P. Acutis, and A. Bagnato. Genetic basis of lipomatous myopathy in piedmontese beef cattle. *Livestock Science*, 206:9–16, 2017.

[49] S. Ponsuksili, E. Murani, M. Schwerin, K. Schellander, D. Tesfaye, and K. Wimmers. Gene expression and dna-methylation of bovine pretransfer endometrium depending on its receptivity after in vitro-produced embryo transfer. 2012.

[50] Y. Ramayo-Caldas, M. Fortes, N. Hudson, L. Porto-Neto, S. Bolormaa, W. Barendse, M. Kelly, S. Moore, M. Goddard, and S. Lehnert. A marker-derived gene network reveals the regulatory role of ppargc1a, hnf4g, and foxp3 in intramuscular fat deposition of beef cattle. *Journal of Animal Science*, 92(7):2832–2845, 2014.

[51] A. Reverter, L. Porto-Neto, M. Fortes, R. McCulloch, R. Lyons, S. Moore, D. Nicol, J. Henshall, and S. Lehnert. Genomic analyses of tropical beef cattle fertility based on genotyping pools of brahman cows with unknown pedigree. *Journal of Animal Science*, 94(10):4096–4108, 2016.

[52] V. M. P. Ribeiro, G. C. Gouveia, M. M. de Moraes, A. E. M. de Araújo, F. S. S. Raidan, P. A. de Souza Fonseca, E. P. Cardoso, M. V. G. B. da Silva, and F. L. B. Toral. Genes underlying genetic correlation between growth, reproductive and parasite burden traits in beef cattle. *Livestock Science*, 244:104332, 2021.

[53] M. Salleh, G. Mazzoni, J. Höglund, D. Olijhoek, P. Lund, P. Løvendahl, and H. Kadarmideen. Rna-seq transcriptomics and pathway analyses reveal potential regulatory genes and molecular mechanisms in high-and low-residual feed intake in nordic dairy cattle. *BMC genomics*, 18(1):1–17, 2017.

[54] C. M. Seabury, D. L. Oldeschulte, M. Saatchi, J. E. Beever, J. E. Decker, Y. A. Halley, E. K. Bhattarai, M. Molaei, H. C. Freetly, and S. L. Hansen. Genome-wide association study for feed efficiency and growth traits in us beef cattle. *BMC genomics*, 18(1):1–25, 2017.

[55] J. Seong, H. Yoon, and H. S. Kong. Identification of microrna and target gene associated with marbling score in korean cattle (hanwoo). *Genes & Genomics*, 38(6):529–538, 2016.

[56] T. Shao, J. C. McCann, and D. W. Shike. Effects of supplements differing in fatty acid profile to late gestational beef cows on steer progeny finishing phase growth performance, carcass characteristics, and mrna expression of myogenic and adipogenic genes. *Animals*, 11(7):1904, 2021.

[57] T. Shimizu, S. Krebs, S. Bauersachs, H. Blum, E. Wolf, and A. Miyamoto. Actions and interactions of progesterone and estrogen on transcriptome profiles of the bovine endometrium. *Physiological genomics*, 42(4):290–300, 2010.

[58] S. Srivastava, K. Srikanth, S. Won, J.-H. Son, J.-E. Park, W. Park, H.-H. Chai, and D. Lim. Haplotype-based genome-wide association study and identification of candidate genes associated with carcass traits in hanwoo cattle. *Genes*, 11(5):551, 2020.

[59] N. B. Stafuzza, E. V. d. Costa e Silva, R. M. d. O. Silva, L. C. C. d. Costa Filho, F. B. Barbosa, G. G. Macedo, R. B. Lobo, and F. Baldi. Genome-wide association study for age at puberty in young nelore bulls. *Journal of Animal Breeding and Genetics*, 137(2):234–244, 2020.

[60] M. S. Tahir, L. T. Nguyen, B. L. Schulz, G. A. Boe-Hansen, M. G. Thomas, S. S. Moore, L. Y. Lau, and M. R. Fortes. Proteomics recapitulates ovarian proteins relevant to puberty and fertility in brahman heifers (bos indicus l.). *Genes*, 10(11):923, 2019.

[61] D. B. A. Teixeira, G. A. F. Júnior, D. B. dos Santos Silva, R. B. Costa, L. Takada, D. G. M. Gordo, T. Bresolin, R. Carvalheiro, F. Baldi, and L. G. De Albuquerque. Genomic analysis of stayability in nellore cattle. *PLoS One*, 12(6), 2017.

[62] P. Tizioto, L. Coutinho, G. Mourão, G. Gasparin, W. Malagó-Jr, F. Bressani, R. Tullio, R. Nassu, J. Taylor, and L. Regitano. Variation in myogenic differentiation 1 mrna abundance is associated with beef tenderness in nelore cattle. *Animal genetics*, 47(4):491–494, 2016.

[63] D. Valour, S. A. Degrelle, A. A. Ponter, C. Giraud-Delville, E. Campion, C. Guyader-Joly, C. Richard, F. Constant, P. Humblot, and C. Ponsart. Energy and lipid metabolism gene expression of d18 embryos in dairy cows is related to dam physiological status. *Physiological genomics*, 46(2):39–56, 2014.

[64] S. M. Waters, G. S. Coyne, D. A. Kenny, and D. G. Morris. Effect of dietary n-3 polyunsaturated fatty acids on transcription factor regulation in the bovine endometrium. *Molecular biology reports*, 41(5):2745–2755, 2014.

[65] R. Whiston, E. K. Finlay, M. S. McCabe, P. Cormican, P. Flynn, A. Cromie, P. J. Hansen, A. Lyons, S. Fair, and P. Lonergan. A dual targeted β-defensin and exome sequencing approach to identify, validate and functionally characterise genes associated with bull fertility. *Scientific reports*, 7(1):1–13, 2017.

[66] P. Widmann, A. Reverter, R. Weikard, K. Suhre, H. M. Hammon, E. Albrecht, and C. Kuehn. Systems biology analysis merging phenotype, metabolomic and genomic data identifies non-smc condensin i complex, subunit g (ncapg) and cellular maintenance processes as major contributors to genetic variability in bovine feed efficiency. *PloS one*, 10(4):e0124574, 2015.

[67] M. R. Wilkens, C. S. Firmenich, N. Schnepel, and A. S. Muscher-Banse. A reduced protein diet modulates enzymes of vitamin d and cholesterol metabolism in young ruminants. *The Journal of steroid biochemistry and molecular biology*, 186:196–202, 2019.

[68] P. Yodklaew, S. Koonawootrittriron, M. A. Elzo, T. Suwanasopee, and T. Laodim. Genome-wide association study for lactation characteristics, milk yield and age at first calving in a thai multibreed dairy cattle population. *Agriculture and Natural Resources*, 51(3):223–230, 2017.

[69] S.-L. Yu, S.-M. Lee, M.-J. Kang, H.-J. Jeong, B.-C. Sang, J.-T. Jeon, and J.-H. Lee. Identification of differentially expressed genes between preadipocytes and adipocytes using affymetrix bovine genome array. *Journal of Animal Science and Technology*, 51(6):443–452, 2009.

[70] F. Zhang, Y. Wang, R. Mukiibi, L. Chen, M. Vinsky, G. Plastow, J. Basarab, P. Stothard, and C. Li. Genetic architecture of quantitative traits in beef cattle revealed by genome wide association studies of imputed whole genome sequence variants: I: Feed efficiency and component traits. *BMC genomics*, 21(1):1–22, 2020.

[71] A. E. Zielak-Steciwko and A. C. Evans. Genomic portrait of ovarian follicle growth regulation in cattle. *Reproductive biology*, 16(3):197–202, 2016.
