# Supplementary material for: Use of a graph neural network to the weighted gene co-expression network analysis of Korean native cattle
Source: Sci Rep. 2022 Jun 14;12:9854. doi: 10.1038/s41598-022-13796-9 (PMC9197844; doi:10.1038/s41598-022-13796-9)
Supplement: Supplementary file 2 — Supplementary Information 2. [file 41598_2022_13796_MOESM2_ESM.pdf]

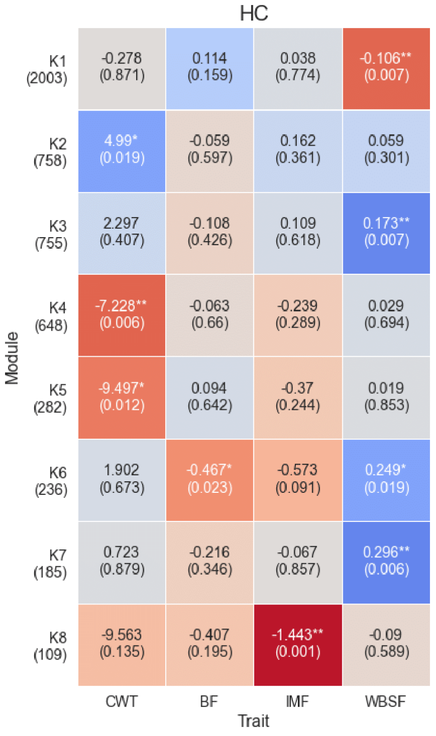

(a)

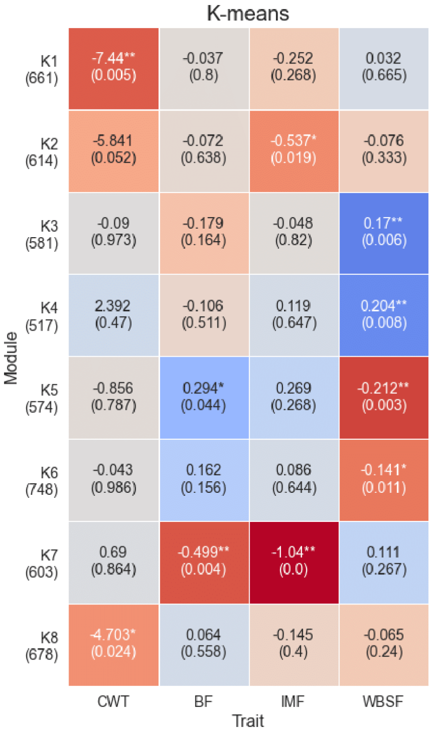

(b)

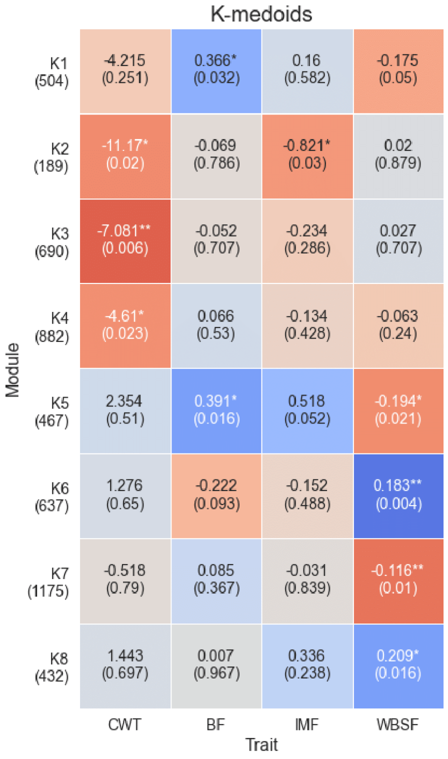

(c)

S1 Fig. The DEM signals of modules defined by baseline methods: (a) hierarchical clustering, (b) K-means, (c) K-medoids. The y-axis shows the module names and numbers of genes within each module. The x-axis shows the complex traits. The numbers in each cell are regression coefficients (no parentheses) and the regression p-values (in parentheses). Red and blue indicate negative and positive coefficients, respectively. * $p < 0.05$, ** $p < 0.01$.
