# Supplementary material for: Use of a graph neural network to the weighted gene co-expression network analysis of Korean native cattle
Source: Sci Rep. 2022 Jun 14;12:9854. doi: 10.1038/s41598-022-13796-9 (PMC9197844; doi:10.1038/s41598-022-13796-9)
Supplement: Supplementary file 3 — Supplementary Information 3. [file 41598_2022_13796_MOESM3_ESM.pdf]

Implementation details for GEO datasets

The NCBI GEO datasets were downloaded from the Gene Expression Omnibus (GEO) repository (https://www.ncbi.nlm.nih.gov/geo/). The datasets include four different species (human: GDS6010, mouse: GDS5618, pig:GDS4246, and chicken: GDS3857). Each dataset includes (human: 18, mouse: 20, pig: 20, chicken: 12) samples with phenotypes (human: virus infection, mouse: pancreatic islets, pig: blood, chicken: light pulse). We excluded non-significant genes to each trait using t-test. Finally, human: 5458, mouse: 6129, pig: 2740, and chicken: 3851 genes were used to this study. The hyper parameters of gmcNet for each dataset are shown in Table 1.

Table 1. The hyper parameters of gmcNet for each dataset.

Dataset	Hyper parameter
Human	k: 4, learning rate: 0.01, m': 8, τ: 0.65, λ: 2.8
Mouse	k: 9, learning rate: 0.01, m': 8, τ: 0.9, λ: 2.8
Pig	k: 6, learning rate: 0.01, m': 8, τ: 0.45, λ: 2.8
Chicken	k: 9, learning rate: 0.01, m': 8, τ: 0.7, λ: 2.4
