# Supplementary material for: Use of a graph neural network to the weighted gene co-expression network analysis of Korean native cattle
Source: Sci Rep. 2022 Jun 14;12:9854. doi: 10.1038/s41598-022-13796-9 (PMC9197844; doi:10.1038/s41598-022-13796-9)
Supplement: Supplementary file 4 — Supplementary Information 4. [file 41598_2022_13796_MOESM4_ESM.pdf]

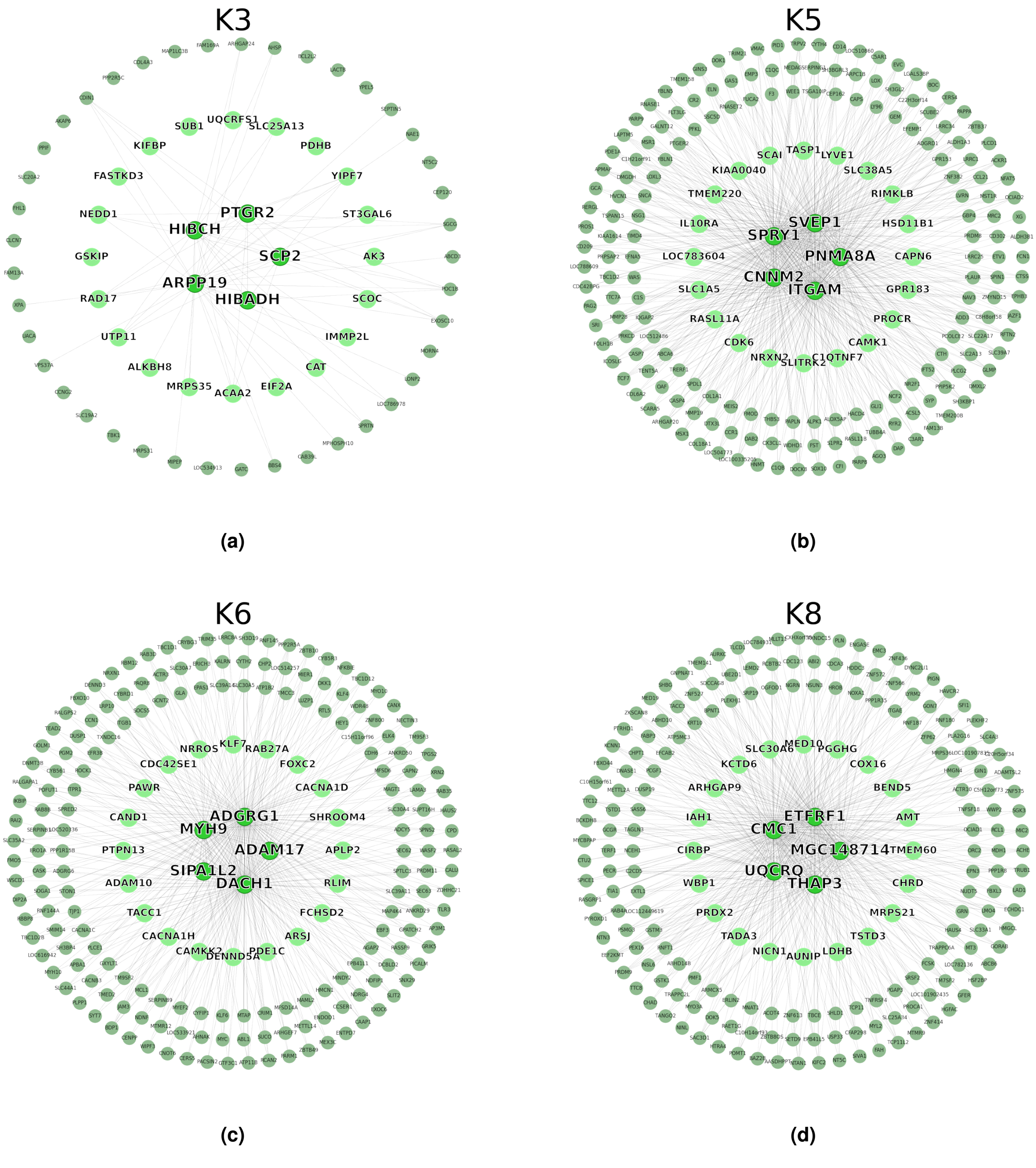

S3 Fig. Hub gene networks of the minor modules: (a) K3, (b) K5, (c) K6, (d) K8. From the outside in, the top 200, top 25, and top 5 hub genes are shown. The linkages of the top 5 hub genes are shown as the edges of the networks.
